# *Trypanosoma cruzi* Infection in Neotropical Wild Carnivores (Mammalia: Carnivora): At the Top of the *T. cruzi* Transmission Chain

**DOI:** 10.1371/journal.pone.0067463

**Published:** 2013-07-04

**Authors:** Fabiana Lopes Rocha, André Luiz Rodrigues Roque, Juliane Saab de Lima, Carolina Carvalho Cheida, Frederico Gemesio Lemos, Fernanda Cavalcanti de Azevedo, Ricardo Corassa Arrais, Daniele Bilac, Heitor Miraglia Herrera, Guilherme Mourão, Ana Maria Jansen

**Affiliations:** 1 Laboratório de Biologia de Tripanosomatídeos, Instituto Oswaldo Cruz, FIOCRUZ, Rio de Janeiro, Brazil; 2 Instituto Brasileiro para Medicina da Conservação, TRÍADE, Recife, Pernambuco, Brazil; 3 Programa de Conservação Mamíferos do Cerrado, PCMC, Araguari, Minas Gerais, Brazil; 4 Programa de Pós-graduação em Ecologia e Conservação, Universidade Federal de Mato Grosso do Sul, UFMS, Campo Grande, Mato Grosso do Sul, Brazil; 5 Programa de Pesquisa em Vida Selvagem/Projeto Carnívoros, Instituto de Pesquisas Cananéia (IPeC), Cananéia, São Paulo, Brazil; 6 Departamento de Ciências Biológicas, Universidade Federal de Goiás, UFG, Catalão, Goiás, Brazil; 7 Departamento de Medicina Preventiva e Saúde Animal, Universidade de São Paulo, USP, São Paulo, Brazil; 8 Universidade Católica Dom Bosco, UCDB, Campo Grande, Mato Grosso do Sul, Brazil; 9 Laboratório de Vida Selvagem, Empresa Brasileira de Pesquisa Agropecuária, Embrapa Pantanal, Corumbá, Mato Grosso do Sul, Brazil; Universidade Federal de Minas Gerais, Brazil

## Abstract

Little is known on the role played by Neotropical wild carnivores in the *Trypanosoma cruzi* transmission cycles. We investigated *T. cruzi* infection in wild carnivores from three sites in Brazil through parasitological and serological tests. The seven carnivore species examined were infected by *T. cruzi*, but high parasitemias detectable by hemoculture were found only in two Procyonidae species. Genotyping by Mini-exon gene, PCR-RFLP (1f8/Akw21I) and kDNA genomic targets revealed that the raccoon (*Procyon cancrivorus*) harbored TcI and the coatis (*Nasua nasua*) harbored TcI, TcII, TcIII-IV and *Trypanosoma rangeli*, in single and mixed infections, besides four *T. cruzi* isolates that displayed odd band patterns in the Mini-exon assay. These findings corroborate the coati can be a bioaccumulator of *T. cruzi* Discrete Typing Units (DTU) and may act as a transmission hub, a connection point joining sylvatic transmission cycles within terrestrial and arboreal mammals and vectors. Also, the odd band patterns observed in coatis’ isolates reinforce that *T. cruzi* diversity might be much higher than currently acknowledged. Additionally, we assembled our data with *T. cruzi* infection on Neotropical carnivores’ literature records to provide a comprehensive analysis of the infection patterns among distinct carnivore species, especially considering their ecological traits and phylogeny. Altogether, fifteen Neotropical carnivore species were found naturally infected by *T. cruzi*. Species diet was associated with *T. cruzi* infection rates, supporting the hypothesis that predator-prey links are important mechanisms for *T. cruzi* maintenance and dispersion in the wild. Distinct *T. cruzi* infection patterns across carnivore species and study sites were notable. Musteloidea species consistently exhibit high parasitemias in different studies which indicate their high infectivity potential. Mesocarnivores that feed on both invertebrates and mammals, including the coati, a host that can be bioaccumulator of *T. cruzi* DTU’s, seem to take place at the top of the *T. cruzi* transmission chain.

## Introduction

The hemoflagellate protozoan *Trypanosoma cruzi* is a multihost parasite that infects mammalian species from eight orders and dozens of triatomine species, the insect vectors [1], [2]. This parasite is the etiological agent of Chagas disease, one of the most important parasitic infections in Latin America [1], [3]. From the distinct *T. cruzi* infection routes, nowadays the oral route has increasing importance due to the high number of oral infection outbreaks in the last decades in Brazil and other Latin American countries [4], [5]. This current epidemiological scenario challenges health authorities because previously employed control measures are not effective against this scenario. Moreover, it emphasizes the importance of looking at the sylvatic cycle to understand all components of this complex system and the environmental factors that underlie the emergence of human cases. Indeed, despite having been recognized by Carlos Chagas as an enzootic parasite already when he described this taxon more than one century ago [6], there are still many open questions regarding the *T. cruzi* ecology and transmission in sylvatic environments.

*Trypanosoma cruzi* exhibits huge biological, biochemical and genetic diversity. Presently, six major genotypes or Discrete Typing Units (DTU) are acknowledged within the taxon, *T. cruzi* I (TcI) to *T. cruzi* VI (TcVI), besides the newly coined TcBat genotype, as yet described as restricted to bats [7]–[9]. Recent studies also reported intraspecific variability within these genotypes, such as TcI isolates [10]. These DTU’s are recognized to be valid units to discriminate *T. cruzi* genotypes, albeit putative associations with vectors and hosts, and the extent of their range are still poorly known. TcI is the most widespread DTU with respect to its geographical distribution [8]. TcII, classically associated to human cases in domestic cycles [8], is also more and more observed infecting sylvatic mammals in several biomes throughout South America [11], [12]. The knowledge concerning TcIII-TcVI is more recent and as new evidence accumulates, we can observe more clearly that the already proposed associations of genotypes with particular hosts, vectors or ecotopes are still under debate and possibly reflect subsampling [11]–[13].

*Trypanosoma cruzi* is maintained in complex transmission nets that occur in overlapping or independent transmission cycles in distinct sylvatic ecotopes [13]–[15]. Although *T. cruzi* is potentially able to infect all mammalian species [16], according to the particularities of host, parasite genotype and their interactions, some species might maintain longer and/or higher parasitemias than others, which will probably reflect different potentials of these hosts to infect vectors and, thus, to serve as reservoirs in a particular intervals of time and space [17]. In this sense, the potential to infect vectors is directly related to the presence of trypomastigotes in the bloodstream of a given mammalian species, thus available to be taken up by the vector during its blood meal. The mammal infection by oral route occurs when the animal ingests infected bug’s feces, food contaminated with the parasite or by preying on infected bugs or mammals [18]. This latter (predator-prey route) can occur through the ingestion of bloodstream trypomastigotes and also by the amastigotes present in the prey tissues as they are capable of infecting host cells [19]–[21]. Another important feature to be accounted is the ecology of each mammalian host species, which might enhance (or not) contact among mammals and vectors that ultimately shape *T. cruzi* transmission dynamics.

Species from the mammalian order Carnivora display a remarkable ecological diversity, having occupied virtually every habitat, vegetational zone and ecological niche. Regardless of their special adaptations for predation, they are widely diverse in their feeding ecology (frugivorous, insectivorous and hypercarnivorous – up to 70% of meat) and likewise in traits such as body size, home range, sociality and activity [22]. Carnivorans were reported to be naturally infected by *T. cruzi* [23] and, considering the efficiency of the *T. cruzi* oral infection route [24], may occupy a unique position as bioaccumulators of parasites and, perhaps, distinct *T. cruzi* DTU’s. Nevertheless, little is known of their role in *T. cruzi* transmission cycles, probably because carnivores are difficult to study as they require specialized management and costly long-term studies. The investigation of *T. cruzi* infection in wild carnivores is essential because this mammalian order exhibits a combination of biological traits that expose themselves to several opportunities of infection by *T. cruzi* in nature: eclectic feeding behavior, long-lived, broad range areas, disperse long distance and the ability to explore both arboreal and terrestrial strata in different habitats. Free-ranging carnivorans, recognized to play a crucial role in regulating ecosystems [25], might also have an important effect in multi-host parasitic transmission webs, like that of *T. cruzi*, as they prey on species from several taxa.

The aim of this study was to determine the role of Neotropical wild carnivores in the *T. cruzi* transmission cycle. Here, we investigated *T. cruzi* infection in the carnivore community of three different sites in Brazil and evaluated the infection patterns in the light of the species ecological traits. We additionally appraised the *T. cruzi* infection records in South America carnivore species to provide a comprehensive analysis of their infection patterns and the possible correlations among distinct carnivore groups (especially considering their habitat, diet and phylogeny). The hypothesis that carnivores are bioaccumulators of *T. cruzi* parasites belonging to distinct DTU’s is also discussed.

## Materials and Methods

### Ethics Statement

This study was approved by the ethics committee of Oswaldo Cruz Foundation/FIOCRUZ (CEUA P-292-06). The capture and sample collection of wild carnivores had permission from the Instituto Chico Mendes de Conservação da Biodiversidade (ICMBio) (SISBIO license number 25078-2 for Pantanal, number 11124-2 for PNSC and number 14576-2 for Araguari/Cumari), in accordance to Brazilian regulations. All animal handling procedures followed the Guidelines of the American Society of Mammalogists [26]. Appropriate biosecurity techniques and individual protection equipment were used during all procedures of collection and handling of the biological samples.

### Study Areas

**Figure 1:**
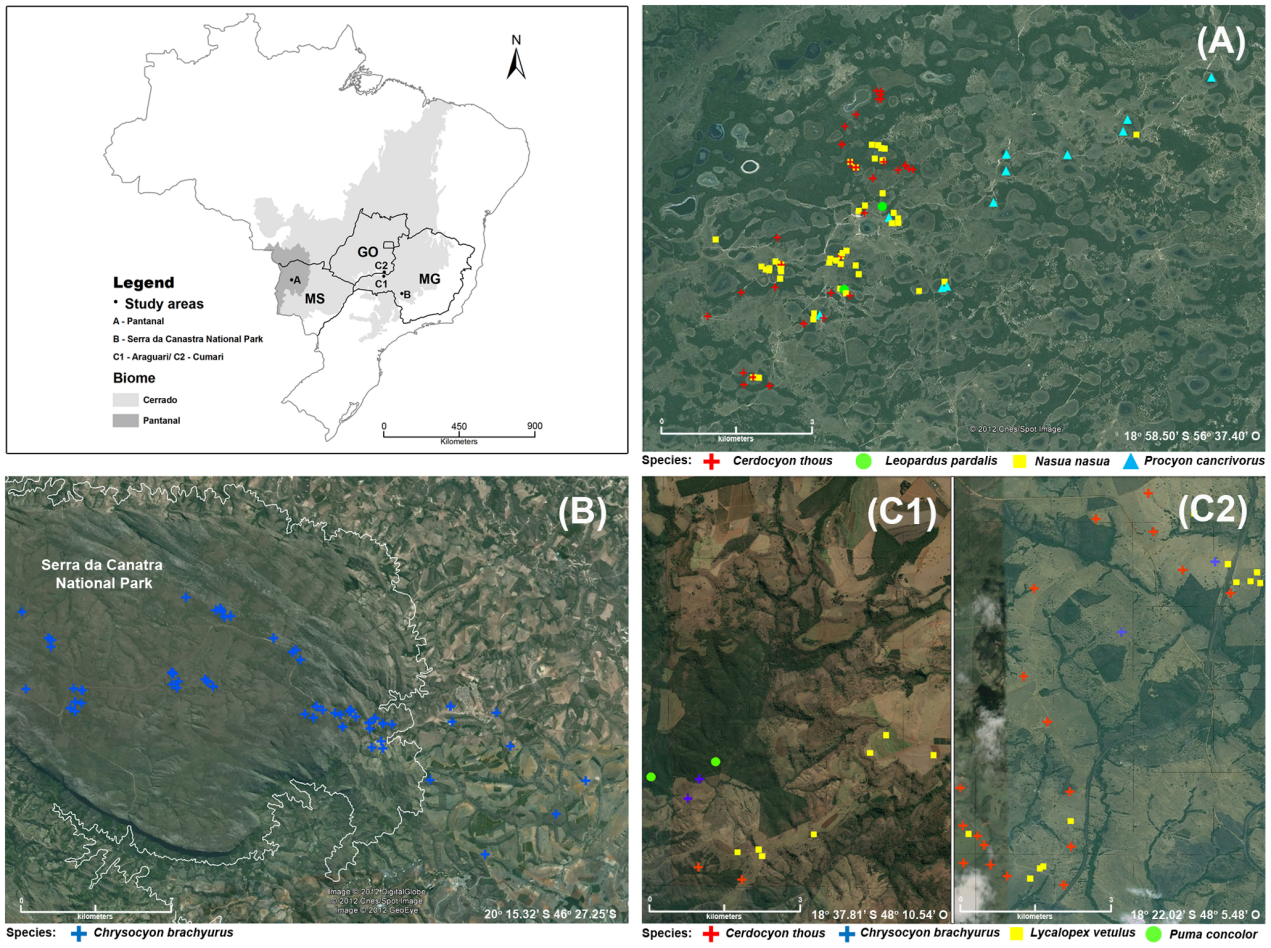
Spatial distribution of free-ranging carnivore species examined for *Trypanosoma cruzi* infection in Brazil. (A) Pantanal - Mato Grosso do Sul State (MS), (B) Serra da Canastra National Park (SCNP) and its surroundings - Minas Gerais State (MG), (C1) Araguari – Minas Gerais State (MG) and (C2) Cumari – Goiás State (GO). Geometric symbols represent carnivore species, according to the figure legend. In the upper left figure the black contour shows the study sites within respective States in Brazil.

Field studies were carried out in three sites in tropical areas of Brazil (Fig. 1). The first site is a research station in the central region of the Pantanal, named Nhecolândia, in the Municipality of Corumbá, Mato Grosso do Sul State (18° 58.50′S 56° 37.40′ W). The Pantanal is a large Neotropical wetland recognized for its abundant and diverse wildlife. The research station is covered by a mosaic of forest patches, savannas, scrub savannas, seasonally flooded grasslands and several permanent or temporary lakes. This area is subjected to annual and multi-annual variations of flooding intensity, with an alternation of high-flood and severe drought years [27]. The second site is the Serra da Canastra National Park – SCNP (20° 15.32′S, 46° 27.75′ W) and surroundings, a conservation unit in the southwest of the Minas Gerais State. The area is a 200,000 hectare remnant of the Cerrado biome, covered with grasslands, interspersed with areas of rocky outcrops, scrub savanna and riparian vegetation. The climate is tropical, the dry season occurs from March to October and the wet season from November to February [28]. The third site comprise extensions within the Municipalities of Araguari, Minas Gerais State (18° 37.81′ S, 48° 10.54′ W) and Cumari, Goiás State (18° 22.02′ S, 48° 5.48′ W). Most of this area is occupied by cattle farms covered with exotic pasture vegetation (*Brachiaria sp.*), although there are still small patches of original Cerrado vegetation. The climate has two well-defined seasons, the wet season from September to March, and the dry season, from April to August [29]. The latter two areas are within the Cerrado biome, which is a vast area of tropical savanna, encompassing about 204 million hectares in the central part of the Brazilian territory [30].

### Carnivore Capture and Sample Collection

Wild carnivores were captured: (*i*) from August 2009 to February 2012 in the Pantanal, except for the ring-tailed coatis, which capture ended in April 2010; (*ii*) from April 2004 to August 2008 in the SCNP region; and (*iii*) from April 2008 to July 2011 in the Araguari/Cumari region. For most carnivore species, we used box traps made with galvanized wire mesh baited with a mixture of bacon, eggs, sardine, fruits, boiled chicken and live chicken according to the target species and study sites. Specifically, pumas (*Puma concolor*) were captured with foot-snares not baited and set along trails previously monitored through camera-traps, while crab-eating raccoons (*Procyon cancrivorus*) were actively searched during night periods and captured using a handheld fishing net. We immobilized the animals with an intramuscular injection of a combination of zolazepan and tiletamine (Zoletil^®^) at dosages of 3–20 mg/kg according to the target species. Anesthetized animals were marked with ear-tags, PIT-tags (passive-integrated-transponder) and/or VHF or GPS radio collars for individual identification. We took blood samples by puncture of the cephalic vein stored in Vacutainer® tubes with EDTA for hemoculture (only in the Pantanal) and serological tests. Animals were released at the site of capture after total recovery from anesthesia. Total capture effort was 2,430 traps-nights in Pantanal, 4,153 traps-nights in SCNP and 1,340 traps-nights in Araguari/Cumari region.

### *Trypanosoma cruzi* Survey

We examined a total of 208 free-ranging carnivores belonging to seven species. The *T. cruzi* infection survey was performed by serological assay and, in the case of the Pantanal, also by hemoculture (HC). This latter was performed as follows: 0.6 ml of blood from each animal was cultured in two tubes (0.3 ml each) containing Novy-Mc Neal-Nicole (NNN) medium with liver infusion tryptose (LIT) overlay. Tubes were examined every fifteen days up to five months. When positive, parasites were grown in LIT, cryopreserved and deposited in the Trypanosomatid collection from wild and domestic animals and vectors - COLTRYP (Oswaldo Cruz Foundation, Rio de Janeiro-RJ, Brazil).

For the detection of anti-*T. cruzi* IgG antibodies in sera, we performed the Indirect Immunofluorescent Antibody Test (IFAT) as described by Camargo [31]. The antigens were prepared using the reference strains F90 (TcI) and Y (TcII) from axenic culture and mixed in equal proportions. Wild canids and felids were tested using domestic dog and cat fluorescein conjugates (SIGMA®), respectively, whereas Procyonidae species were tested by IFAT using a goat anti-raccoon IgG fluorescein conjugate (KPL®). We adopted an IFAT cut-off value of 1/40 for all species, due to the possibility of cross-reaction with other trypanosomatid parasites. Each reaction included two positive and two negative control sera.

In order to detect possible dual infection or cross-reaction with *Leishmania* spp. sera were also tested by IFAT test using *L. infantum* (IOC/L579– MHOM/BR/1974/PP75) and *L. braziliensis* (IOC/L566– MHOM/BR/1975/M2903) promastigotes obtained from the Collection of Leishmania from Oswaldo Cruz Institute (CLIOC) mixed in equal proportion as antigens (cut-off: 1∶40). Wild canids were additionally tested for *Leishmania* infection using the Rapid Test for Diagnosis of Canine Visceral Leishmaniasis (TR DPP®, BioManguinhos, Rio de Janeiro, Brazil) and the Enzyme-Linked Immunoabsorbent Assay (ELISA, Biomanguinhos, Rio de Janeiro-RJ, Brazil). Cut-off value for ELISA was optical absorbance ≥0.200 mean ±3 SD. If positive for *Leishmania* infection in IFAT, DPP or ELISA, samples were considered positive to *T. cruzi* only when the IFAT titer for *T. cruzi* was 1/80 or higher.

For the seroprevalence calculation, each individual was counted once (positive/total number of sampled individuals). For the hemoculture prevalence, we incorporated all samples, including recaptured individuals.

Seropositive animals that had negative results in the parasitological test were considered to be infected with *T. cruzi* but with low parasitemia, thus probably less prone to be infective to the vectors. Parasitological tests, such as hemoculture, xenodiagnosis or fresh blood examination, are less sensitive and when positive reflect high parasite burden. Thus, positive hemocultures demonstrate their transmissibility potential, *i.e*., the potential of these hosts to infect vectors.

### *Trypanosoma cruzi* Molecular Characterization

Epimastigote forms collected from positive hemocultures at the end of the log phase were washed, and incubated with proteinase K and SDS (sodium dodecyl sulfate). The genomic DNA of the lysed cells was extracted with standard phenol-chloroform protocols [32]. Characterization was carried out by multiplex PCR amplification of the non-transcribed spacer of the mini-exon gene (MMPCR) [33]. DTU's were identified according to amplicon size: 150 bp (TcIII/TcIV), 200 bp (TcI) and 250 bp (TcII/TcV/TcVI), besides *T. rangeli* – TR (100 bp) [34]. When MMPCR resulted in TcII/TcV/TcVI band pattern we performed a PCR amplification of the nuclear 1f8 gene followed by restriction fragment length polymorphism (RFLP) analysis of fragments digested by Alw21I enzyme [35] to discriminate TcII from hybrids (TcV and TcVI). We additionally performed kDNA PCR amplification [32], which distinguishes *T. rangeli* (a specific 760 bp band besides variable fragments of 300–450 bp) from *T. cruzi* (only one 330 bp band) in order to confirm *T. cruzi* in isolates that could not be characterized with the previous genomic targets. Each reaction included sterile distilled water instead of DNA as a negative control and positive control samples from *T. cruzi* strains representing the DTUs. PCR products were visualized under ultraviolet light after electrophoresis in 2% agarose gel with ethidium bromide staining.

### Statistical Analysis

We used chi-square tests (α = 0.05) to investigate significant differences between seroprevalence rates for the different species and host sex in each study area.

In order to determine if *T. cruzi* infection rates in wild carnivores was associated with species diet, we retrieved data on the diet of the different species from the literature, taking into account the study site. The only exception was for the crab-eating raccoon, as there are no studies on its diet performed in the Pantanal. We first made scatter diagrams of the proportion of invertebrates in the species diet and the *T. cruzi* exposure rates to visualize the mathematical function that best represented the relationship between these variables. From the graphic analysis we estimated the linear regression model. Species with n <10 specimens per study site were excluded from this analysis. The *T. cruzi* exposure rates, hosts and diet assigned for each species, besides the references used are shown in supporting information (Table S1).

To test if infectiveness rates (the potential of the host to infect vectors as demonstrated by positive hemoculture, xenodiagnosis or fresh blood examination) between different taxa were phylogenetically autocorrelated we used the phylogenetic topology of Agnarsson et al. [36] to compute proximity matrices. For this, we calculated the infection rate (%) for each species as the total positive/total examined * 100. A complete list of data sets and source references is shown in supporting information (Table S2). We used Moran's autocorrelation index [37], Abouheif's test [38] with Abouheif's original matrix proximity calculus, Thioulouse et al. [39] implementation and patristic distances. Statistical significance of tests was done using 1,000 MonteCarlo permutations. We also tested for the phylogenetic effect of infectivity using ANOVA on phylogenetic eigenvector decomposition of the phylogenetic tree [40]. All computations were done under R 2.13 software and used packages ade4 [41], adephylo [42], ape3.0–5 and phylobase [43].

## Results

### Pantanal

The four examined carnivore species, the ocelot (*Leopardus pardalis*), the crab-eating fox (*Cerdocyon thous*), the ring-tailed coati (*Nasua nasua*) and the crab-eating raccoon (*Procyon cancrivorus*), were infected by *T. cruzi* as demonstrated by the high rates of positive IFAT tests, but high parasitemias, expressed by positive hemocultures, were found only in the two Procyonidae species (Table 1).

**Table 1:** *Trypanosoma cruzi* infection assessment of wild carnivores from three study sites in Brazil and ecological data.

Species (common name)	SexF/M	*T. cruzi* infection				Ecological data						
		Serology(IFAT)		Hemoculture		Diet	PM(%)	PI(%)	Activity	Strata	Habitattype	Ecological datareferences^c^
		P/T (%)^a^	F/M^b^	P/T^a^ (%)	F/M^b^							
**STUDY SITE: PANTANAL**												
*Cerdocyon thous* (crab-eating fox)	14/16	19/30 (63)	9/10	0/30 (0)	–	C/F/I	32.8	33.4	C_N	T	Scrub/Savanna	[52]
*Leopardus pardalis* (Ocelot)	2/0	2/2 (100)	2/0	0/2 (0)	–	C	93.5	6.7	N	T	Forest	[52]
*Nasua nasua* (ring-tailed coati)	26/40	21/44 (48)	10/11	19/66 (29)	6/13	C/F/I	14.3	46.6	D	T/S	Scrub/Forest	[52]
*Procyon cancrivorus* (crab-eating raccoon)	6/7	9/12 (75)	4/5	2/13 (15)	1/1	C/F/I	3.8	41.7	N_C	T	Grassland/Forest	[65]
**STUDY SITE: SERRA DA CANASTRA NATIONAL PARK**												
*Chrysocyon brachyurus* (maned wolf)	24/19	11/43 (26)	7/4	–	–	C/F	24.4	4.3	C_N	T	Grassland	[66], [67]
**STUDY SITE: ARAGUARI/CUMARI**												
*Cerdocyon thous*	13/14	9/27 (33)	5/4	–	–	C/F/I	17.5	25.9	C_N	T	Scrub/Savanna	[67], [28]
*Chrysocyon brachyurus*	2/2	0/4 (0)	0/0	–	–	C/F	24.4	4.3	C_N	T	Grassland	[66], [67]
*Lycalopex vetulus* (hoary fox)	10/11	15/21 (71)	8/7	–	–	C/F/I	5.5	70.4	N	T	Grassland	[67], [28]
*Puma concolor* (puma)	0/2	2/2 (100)	0/2	–	–	C	80	–	N	T	Scrub/Savanna	[68], [69]

Footnotes:

IFAT - Indirect Immunofluorescent Antibody Test.

a Positive/Total number of examined (% positive).

b Positive female/male.

Species diet (C - carnivorous; F-frugivorous; I-insectivorous; PM proportion of mammals; PI proportion of invertebrates).

Activity (C crepuscular, N nocturnal, D diurnal), strata occurrence (T, terrestrial; S scansorial).

c The ecological data was retrieved from the literature.

The two ocelots examined tested positive in the IFAT (titers 1∶80 and 1∶160) and the other three carnivore species were similarly exposed to *T. cruzi* infection (range 48–75%), as no significant differences was found in the seroprevalence rates among them (χ*^2^* = 3.62, *df = *2, *p*<0.16). The crab-eating fox seroprevalence was 63% and titers did not exceed 1∶80. The seroconversion observed among 7 out of 8 recaptured individuals, pointed to the existence of an active *T. cruzi* transmission cycle. For the two Procyonidae species, the seroprevalence were 48% and 75%, for the ring-tailed coati and crab-eating raccoon, respectively, with titers reaching up to 1∶320. Gender difference in seroprevalence was observed only in the ring-tailed coati, being higher in females (77%) than males (35%) (χ*^2^* = 6.3; *df = *1, *p*<0.01).

The prevalence of positive HC was nearly two times higher for ring-tailed coatis (29%) than for crab-eating raccoons (15%). Moreover, one ring-tailed coati was HC positive in the first capture and also in the second capture 14 months later.

We obtained 20 *Trypanosoma sp.* isolates morphologically similar to *T. cruzi* and *T. rangeli* through hemoculture, of these, three (two from ring-tailed coatis and one from crab-eating raccoon) could not be characterized due to contamination during field procedures. Genotyping from the 16 ring-tailed coati isolates revealed that twelve of them were TcI in single infections (n = 6, 37.5%) and mixed infections: TcI and TcII/TcV/TcVI (n = 2, 12.5%), TcI and TcIII/IV (n = 1, 6.3%) or TcI and *Trypanosoma rangeli* (n = 3, 18.8%). The single characterized isolate from the crab-eating raccoon was genotyped as TcI.

**Figure 2:**
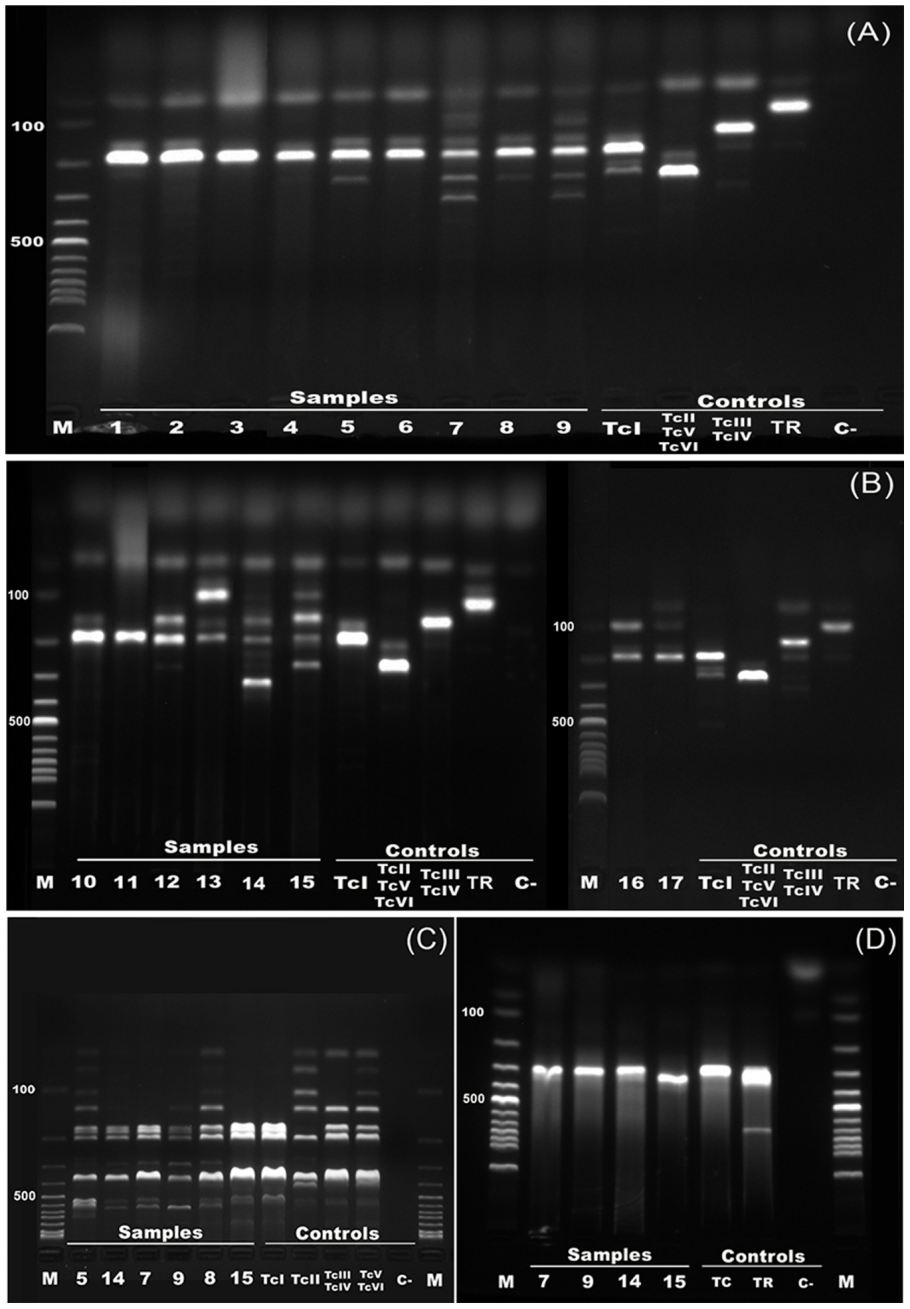
*Trypanosoma cruzi* genotyping of wild carnivore isolates from the Pantanal wetland, Brazil. Representative agarose electrophoresis gels stained with ethidium bromide of the (A and B) Mini-exon multiplex PCR products, (C) 1f8 gene/Alw21I PCR-RFLP products and (D) kDNA PCR products. Lanes: M. Molecular weight markers (100bp DNA ladder), 1- *Procyon cancrivorous* isolate; 2–17– *Nasua nasua* isolates. Control samples: TcI, TcII, TcIII/TcIV, TcV/TcVI, TR - *T. rangeli*, TC – *T. cruzi* and C- Negative control.

We were unable to define the *T. cruzi* genotype in four ring-tailed coati’s isolates (numbers 7, 9, 14, 15– Fig. 2). The MMPCR characterization on these isolates resulted on multiple bands pattern across repeated experiments, including an odd band about 320bp (isolates 7, 9 and 14). The 1f8/Alw21I assay confirmed only DTU TcI. The kDNA assay confirmed the four isolates were *T. cruzi*.

### Serra Da Canastra National Park (SCNP)

An active *T. cruzi* transmission cycle among carnivores was also observed in the maned wolf (*Chrysocyon brachyurus*) population from the SCNP region. Seroprevalence was 26% and among non-infected recaptured individuals (n = 10), 4 seroconverted after one-year follow-up and other 2 seroconverted after two years. In all cases, titers did not exceed 1∶80. No significant differences was found between genders (χ*^2^* = 0.36; *df = *1, *p*<0.54).

### Araguari/Cumari

In this region none of the maned wolves (n = 4) displayed positive serological tests and we found a significative difference in the *T. cruzi* infection rates among the other three examined species (χ*^2^* = 8.79, *df = *2, *p*<0.01) (Table 1).

The two puma specimens tested positive in the IFAT (titers - 1∶40 and 1∶160). In the two canid species, the hoary fox’s (*Lycalopex vetulus*) seroprevalence rate (71%) was two times higher than that of the crab-eating fox’s (33%). Titers for both species reached up to 1∶320. Neither species had differences in seroprevalence amid gender (hoary fox: χ*^2^* = 0.68; *df = *1, *p* = 0.40; crab-eating fox: χ*^2^* = 0.29; *df = *1, *p* = 0.58).

### *Trypanosoma cruzi* Infection Rates in Relation to Species Diet

**Figure 3:**
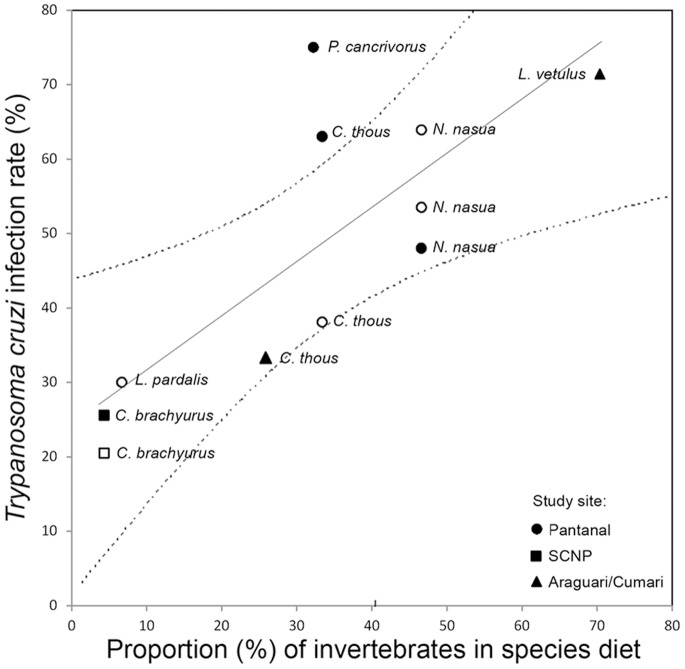
*Trypanosoma cruzi* infection in Neotropical wild carnivores and the proportion of invertebrates in species’ diet. Infection rate (total examined/total positive*100) was determined by IFAT - Indirect Immunofluorescent Antibody Test. Studies sites were Pantanal – Mato Grosso do Sul State, Araguari - Minas Gerais State/Cumari – Goiás State and Serra da Canastra National Park (SCNP) – Minas Gerais State, Brazil. The data set included samples collected on this study (filled symbols) and from the same studies sites previously published by our group. Species’ diets were retrieved from the literature (Table S1). The fitted linear regression (F(1,9): 14.9; r^2^ = 0.62, p = 0.004) is represented by the solid line (y = 0.73x+24). Dashed lines indicate the confidence intervals at 95%.

T. cruzi infection rates in wild carnivores significantly increased with the proportion of invertebrates in the species diet (r^2^ = 0.623; df = 9; p<0.004; Fig. 3). In addition, the four felids examined, which include a high proportion of mammals in their diet, were all infected (Table 1).

### Neotropical Wild Carnivores as *Trypanosoma cruzi* Host

Data on Neotropical wild carnivore *T. cruzi* hosts combining our results and literature records were assembled in Table 2. Fifteen Neotropical carnivore species belonging to five families were described to be infected by *T. cruzi* by serological and/or parasitological methods in Argentine, Brazil, Chile and Colombia.

**Table 2:** Neotropical wild carnivores naturally infected by *Trypanosoma cruzi* from this study (in bold) and literature records.

Species^a^	Serological	Parasitological	Lineage	State/Country [references]
	P/T (%)^b^	P/T (%)^b^	(DTU)^c^	
**FAMILY CANIDAE**				
***Cerdocyon thous***	**9/27 (33)**	–	–	**Goiás-Minas Gerais/BR**
***Cerdocyon thous***	**18/30 (60)**	**0/30 (0)**	–	**Mato Grosso do Sul/BR**
*Cerdocyon thous*	16/42 (38)	0/42 (0)	–	Mato Grosso do Sul/BR [^14^]
*Cerdocyon thous*	4/8 (50)	0/3 (0)	–	Minas Gerais/BR [^15^]
*Cerdocyon thous*	–	1/5 (20)	–	São Paulo/BR [^70^]
***Chrysocyon brachyurus***	**11/43 (26)**	–	–	**Minas Gerais/BR**
*Chrysocyon brachyurus*	8/39 (21)	0/30 (0)	–	Minas Gerais/BR [^15^]
*Lycalopex culpaeus*	–	4/77 (5)	–	CL [^71^]
*Lycalopex culpaeus*	–	1/15 (7)	–	Freirina/CL [^72^]
*Lycalopex culpaeus*	–	1/2 (50)	–	Jujuy/AR [^73^]
*Lycalopex griseus*	–	2/29 (7)	–	Freirina/CL [^72^]
*Lycalopex gymnocercus*	–	1/1	–	Salta/AR [^73^]
***Lycalopex vetulus***	**15/21 (71)**	–	–	**Goiás-Minas Gerais/BR**
*Lycalopex vetulus*	–	1/1	–	São Paulo/BR [^59^]
**FAMILY FELIDAE**				
***Leopardus pardalis***	**2/2 (100)**	**0/2 (0)**	–	**Mato Grosso do Sul/BR**
*Leopardus pardalis*	3/10 (30)	0/3 (0)	–	Mato Grosso do Sul/BR [^14^]
*Leopardus pardalis*	1/1	1/1	TcI	Minas Gerais/BR [^15^]
***Puma concolor***	**2/2 (100)**	–	–	**Goiás-Minas Gerais/BR**
**FAMILY MEPHITIDAE**				
*Conepatus chinga*	–	1/15 (6.6)	TcIII*^c1^*	Santiago del Estero/AR [^74^]
*Conepatus chinga*	–	1/91 (1.1)	TcIII*^c1^*	Santiago del Estero/AR [^75^]
*Conepatus chinga*	–	2/36 (5.5)	–	Santiago del Estero/AR [^76^]
*Conepatus chinga*	–	2/49 (4.1)	–	Santiago del Estero/AR [^77^]
**FAMILY MUSTELIDAE**				
*Eira barbara*	–	1/5 (20)	–	São Paulo/BR [^78^]
*Eira barbara*	–	1/2 (50)	–	Mato Grosso/BR [^79^]
*Eira barbara*	–	2/4 (50)	–	Pará/BR [^80^]
*Eira barbara*	–	1/4 (25)	–	Pará/BR [^81^]
*Eira barbara*	–	1/1	–	Jujuy/AR [^73^]
*Galictis cuja*	–	1/1	–	Santiago del Estero/AR [^76^]
*Galictis cuja*	–	2/14 (14)	–	São Paulo/BR [^82^]
*Galictis vittata*	–	1/1	–	São Paulo/BR [^83^]
*Galictis vittata*	–	1/1	TcII/TcV/TcVI*^c2^*and TcIII/IV*^c3^*	Rio de Janeiro/BR [^84^]
**FAMILY PROCYONIDAE**				
***Nasua nasua***	**21/44 (48)**	**19/66 (29)**	**TcI, TcII, TcIII/TcIV**	**Mato Grosso do Sul/BR**
*Nasua nasua*	75/140 (54)	53/140 (38)	TcI and TcII/TcV/TcVI*^c2^*	Mato Grosso do Sul/BR [^45^]
*Nasua nasua*	101/158 (64)	33/158 (21)	TcI and TcII/TcV/TcVI*^c2^*and TcIII/TcIV*^c3^*	Mato Grosso do Sul/BR [^44^]
*Nasua nasua*	–	7/18 (39)	TcIII/TcIV*^c3^*	Pará/BR [^85^] [^86^]
*Nasua nasua*	–	1/5 (20)	–	São Paulo/BR [^87^]
*Potus flavus*	–	1/2 (50)	TcI	Bajo Colima/CO [^34^], [^88^]
***Procyon cancrivorus***	**9/12 (75)**	**2/13 (15)**	**TcI**	**Mato Grosso do Sul/BR**
*Procyon cancrivorus*	–	1/4 (25)	–	São Paulo/BR [^89^]

Footnotes:

(−) Not available.

Serological test: IFAT - Indirect Immunofluorescent Antibody Test.

Parasitological tests: Hemoculture, xenodiagnosis or fresh blood examination.

a We adopted Wilson & Reeder [90] for taxonomic reference; thus, host species names reported in this table not always correspond to the original paper.

b Positive/Total number of examined (% positive).

Countries: AR – Argentine, BR – Brazil, CL – Chile, CO – Colombia.

c Current nomenclatural consensus as Discrete Typing Units (DTU) following Zingales et al. [7]. Original classification and equivalence to currently grouping scheme. *^c1^*TCIIc = TcIII; *^c2^* TcII (Mini-exon gene) = TcII/TcV/TcVI, *^c3^* Z3 = TcIII/TcIV.

Distinct *T. cruzi* infection patterns among carnivore species were notable. Procyonids demonstrated to have a high potential to infect vectors, in particular the ring-tailed coati, given the persistently high parasitological prevalence rates (Table 2). Also, this species was recorded harboring the main *T. cruzi* lineages in single and mixed infection, corroborating its bioaccumulator potential. In spite of the few data available, mustelids might also display high potential to infect vectors and one species was recorded harboring mixed infections. Regarding the Mephitidae family, one single species, the skunk (*Conepatus chinga*) was found infected: parasitological tests consistently tested positive and this species harbored TcIII in single infections in Argentina. In canid species, high parasitemias were observed only in a few individuals of the *Lycalopex* genus and one crab-eating fox. The *T. cruzi* infection in felids is poorly reported: one ocelot was reported to have high parasitemia and the serological detection of *T. cruzi* infection in pumas suggests that they may also be involved in the *T. cruzi* network (Table 2).

**Figure 4:**
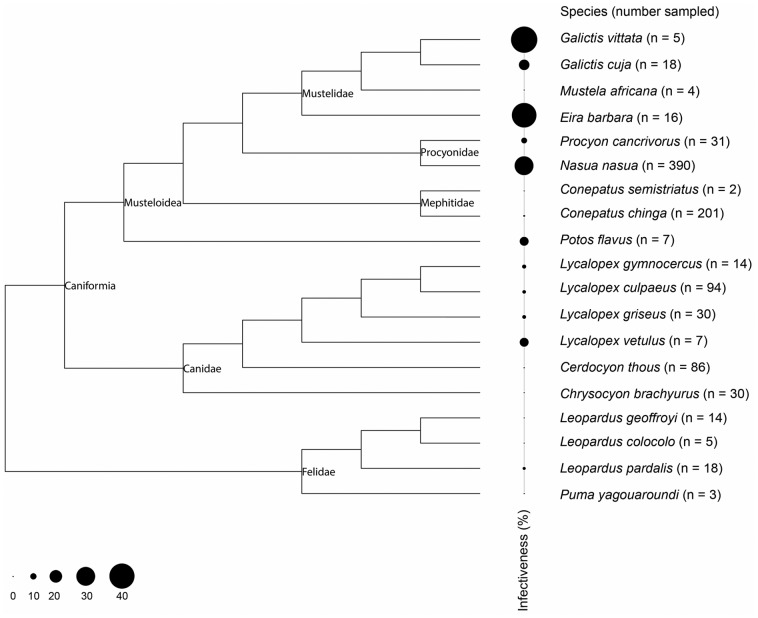
*Trypanosoma cruzi* infectiveness rates in Neotropical carnivore species at the tips of their phylogeny. The size of circles denotes the infectiveness rates, which were determined as the total positive/total examined*100 in hemoculture or xenodiagnosis tests. Phylogenetic topology of Agnarsson et al. [36].

The aforementioned differences in infection pattern among carnivore species were not phylogenetically autocorrelated, as none of the tests revealed a significant influence of carnivore phylogeny on *T. cruzi* infection detected by hemoculture/xenodiagnosis assays. For Moran's test I = 0.059, and the expected null value was −0.055 (p = 0.198). Abouheif's test with different proximity matrices were also not significant even though patristic distance retrieved marginally significant results (p = 0.077 – original; p = 0.099 – Thioulouse, p = 0.044 - patristic). The global ANOVA of infectiveness on phylogenetic structure was not significant (p = 0.277). However, tests on each of the 7 eigenvector showed that the first phylogenetic eigenvector, which contrasts the superfamily Musteloidea vs. families Canidae and Felidae was significant (p = 0.02689, Fig. S1). This indicates a difference in infectiveness rates between these groups (Fig. 4).

## Discussion

Fourteen Neotropical carnivore species belonging to five families were already described to be infected by *T. cruzi* in Argentine, Brazil, Chile and Colombia. The fifteenth one, the puma was found infected in the present study. Distinct *T. cruzi* infection patterns across carnivore species and study sites were notable, as also observed among the seven species herein studied in three different areas of Brazil. Particularly interesting is the finding that carnivore species, mainly procyonids and mustelids, often exhibited high parasitemias and were able to harbor the main *T. cruzi* genotypes, both in single and mixed infections. We also evidenced that species diet could influence the *T. cruzi* infection rates.

In the Pantanal the *T. cruzi* cycle included all examined carnivore species, as previously reported in the same study site [14]. Notwithstanding, based on the positive hemoculture only the ring-tailed coatis and the crab-eating raccoons demonstrated a potential to infect vectors. This is in accordance with previous studies that report the role played by the ring-tailed coati as the main reservoir of *T. cruzi* in the Pantanal due to its high prevalence of infection, long-lasting parasitemia and infection with the main *T. cruzi* lineages [14], [44], [45]. Moreover, the coati has numerous paths for contact with *T. cruzi*, as it occupies both arboreal and terrestrial strata in several habitat types and can prey on infected insects and mammals [46]. Another coati’s ecological characteristic that facilitates infection by *T. cruzi* is its behavior of construction of arboreal nests for resting and reproduction, as we have found several of these nests infested with triatomine bugs in our recent field observations (J. Saab, *unpublished data*). Along with its potential to infect vectors and the capacity to maintain multiples lineages of the parasite [44], [45], this species may be considered a network hub, a common connection point joining transmission cycles maintained within terrestrial and arboreal mammals and vectors.

We observed a temporal change in the ratio of infection by the different *T. cruzi* lineages in the coati population of the Pantanal in comparison to former reports. Previously, single TcI and TcII/TcV/TcVI infections were reported to occur in similar ratios, whereas TcIII/TcIV (formerly Z3) and *Trypanosoma rangeli* (TR) occurred in minor proportion [44]. Herein, TcI has prevailed over TcII/TcV/TcVI whereas TcIII/TcIV, which was not reported in the past few years [45], was isolated again from the coati population. We also observed a gradual increase in the prevalence of *T. rangeli* (3%–[44]; 7%–[45]; 20% this study). It is well-known that the striking variation of the flooding intensity in the Pantanal, as well as other environmental changes, has a strong effect on the local community structure, modulating vectors, hosts and consequently their parasites density, distribution and interrelationships [27], [47]. In the same manner changes in the hosts’ environment are expected to impact the parasite subpopulation (in this case, *T. cruzi* DTU’s), but the outcome of this impact might be different according to each subpopulation dynamics. For instance, TcI infects a huge variety of mammal host species and its infection is described as resulting in high and long lasting parasitemias [8], [17], [48]. This is not the case of TcII and probably may also not be the case of TcIII/TcIV (formerly Z3), which infection results in short period parasitemias and are described to occur in more restricted transmission cycles [8], [17]. Therefore the distribution of TcI in a given environment is expected to be less impacted by environmental changes than TcII and TcIII/TcIV (formerly Z3). Likewise with any other living being populations, *T. cruzi* subpopulations might also expand and retract according to the resources available. Moreover, competitive exclusion and interactions between tripanosomatid species and/or *T. cruzi* subpopulations certainly take a role in modulating the ratios of infection. In any case, this highlight how complex and dynamic the *T. cruzi* transmission cycles can be, even regarding a single species in a small snippet of time and space.

We were not able to define the *T. cruzi* genotype in four out of 16 ring-tailed coati isolates, as they displayed multiple bands, besides and additional odd band pattern of 320bp in the MMPCR assay. The other two molecular targets employed confirmed only TcI and excluded *T. rangeli*, showing that the band pattern observed was not related to multiple infections. In this sense, we are probably facing *T. cruzi* isolates with odd band patterns in the MMPCR assay, a method routinely used worldwide and recently validated for a rapid typing of *T. cruzi* DTU groups [34]. Indeed, unusual profiles within different molecular targets, including the mini-exon gene, had already been observed in *T. cruzi* wild mammal isolates [49]. Rather than methodological constraints, these observations may point out that any time we might deal with undiscovered/unusual lineages which the most employed markers are not capable of characterizing, especially while working with isolates derived from wild hosts. These findings reinforce that the diversity of *T. cruzi* might be much higher than currently acknowledged, as evidenced, for example, by the recent description of the Tcbat [50].

As already observed [45], the ring-tailed coati was the only carnivore species examined in this study to display gender differences in the infection rate. Coatis have an unique social system among carnivore species; males and females have marked differences in their ecology, as females live in groups while most adult males are solitary (except during the breeding season) [51]. The rate of infection by *T. cruzi* in a specific host is driven by contact processes among vector-parasite-host; thus we might expect that these ecological dissimilarities lead to different infection ratios. In the case of ring-tailed coatis, females were observed to have differential habitat use during reproductive period [52] and to spend more time in nests, whereas males’ coatis are not involved in parental care [53]. This may put females at greater risk for exposure to *T. cruzi*, as these nests are a suitable ecotope for triatomines (J. Saab, *unpublished data*), indicating that habitat use and not gender is the deciding factor here.

The crab-eating raccoon was another Procyonidae species implicated in the maintenance and dispersion of *T. cruzi* in the Pantanal region. Different from the congener common raccoon *Procyon lotor,* widely studied and the most commonly reported *T. cruzi* host in North America (along with *Didelphis virginiana*) [54], the crab-eating raccoon is poorly studied. This raccoon species is nocturnal, omnivorous and highly associated with water. Shelter sites in Pantanal were frequently registered in clusters of terrestrial bromeliad (*Bromelia balansae*) (C. Cheida, *unpublished data*), a recognized habitat for the vector species *Triatoma sordida* and *Pastrongylus megistus*, both known to occur in the Mato Grosso do Sul State [55]. The high parasitemias observed in this study corroborate that the crab-eating raccoon, just as the other procyonids, has a potential to infect triatomines.

The crab-eating foxes and the ocelots from the Pantanal, although highly exposed, did not display high parasitemias. Regarding the crab-eating foxes, our hypothesis is that the pattern of *T. cruzi* infection in this species, and probably in other wild canid species, is similar to that found in domestic dogs, which is characterized by a short period of patent parasitemia during the acute phase, followed by a chronic phase with low recovery of positive hemocultures, even in reinfections [56]. Also, in spite of its plasticity in diet and habitat preferences [29], this species has much less capacity to explore microhabitats suitable for triatomines than the coatis with its great ability with its paws and snout [57]. Besides, differently from coatis, the crab-eating fox are restricted to the terrestrial environment. On the other hand, the ocelot has the most carnivorous diet among these three species and can explore both terrestrial and arboreal strata. In such hypercarnivorous, *T. cruzi* infection might reflect the rates of infection among their prey [15]. Indeed, ocelots from the Pantanal tested negative in hemoculture and the small mammals from the same region were reported to display low HC prevalence [58].

A *T. cruzi* transmission cycle involving another carnivore species, the maned wolf, was also observed in the Serra da Canastra National Park region. Our results on *T. cruzi* infection in maned wolves verified by serology are in accordance with a previous study conducted by us [15] and both reported seroconversion. Together, this eight-year follow-up attested that *T. cruzi* is enzootic among maned wolves from SCNP, and also that transmission is well-established and active in the area.

In Araguari/Cumari region, the two tested puma were infected by *T. cruzi*, possible due to the top chain position of this species, besides the classical contaminative route. Indeed, to the best of our knowledge, this is the first such report from a top predator. Infection rates in the hoary fox was two times higher than the infection rates in the crab-eating fox, and the other canid species examined, the maned wolf, tested negative. The present study represents the first sizable data on the hoary fox since a single specimen was previously described infected in the early 70′s [59]. Amongst those three canids, the hoary fox seems to be the most likely to be exposed to triatomine bugs due to their ecological characteristics. It is the only Neotropical canid with a predominantly insectivore diet and also the only of those three canids to use armadillo burrows regularly [29], [60], a recognized ecotope for some vectors in this area, such as the *Panstrongylus* species [55], [61].

The oral transmission has been constantly suggested as the principal mechanism of parasite dispersion among free-ranging mammals and recently, even humans [5], [62]. This route may be of particular importance for carnivore species, since many of them feed on both insects and mammals [63], thus providing several opportunities for infection throughout its life. The classic contaminative route may also be present in some of these infections, but it is probably less prone to be the most effective because some of these carnivores are very active at night, do not use permanent dens, and the contaminated feces still would have to pass the usually dense fur of these carnivorans. Herein, we demonstrated that the higher the proportion of invertebrates in species diet, the greater *T. cruzi* infection rate. Interestingly, the *T. cruzi* exposure rate in the three canid species, the hoary fox, the crab-eating fox and the maned wolf, scales in the proportion of the amount of invertebrates in their diet. The transmission by the ingestion of infected triatomines is highly efficient and was already demonstrated in the carnivores striped skunk (*Mephitis mephitis*) [64] and raccoon (*Procyon lotor*) [21]. Our results suggest that carnivore species with insectivorous diet would have more probability of contact with triatomine bugs while foraging. For top-chain species, the infection through the ingestion of infected prey might also be an important infection route [19].

Our assemblage on *T. cruzi* infection among Neotropical carnivore species corroborated epidemiological evidence that they are involved in the *T. cruzi* transmission networks, though with variable potential to amplify and disperse parasite populations according to the species ecological characteristics and regional peculiarities. The long-term maintenance of the *T. cruzi* transmission cycles will rely on vector feeding upon mammals displaying patent parasitemias. Herein, we found that species belonging to the superfamily Musteloidea were more likely to display high parasitemias and thus to infect vectors. One common component within this group is that, despite the adaptations to predation of vertebrates as other carnivore species, many species are omnivores [22]. Mesocarnivores feed on both invertebrates and mammals and thus seems to take a place at the top of the *T. cruzi* transmission chain. In fact, the frequent findings of mixed infection, as demonstrated by the coati, corroborate the bioaccumulator potential expected for mammals at the top of the parasite chains.

## Supporting Information

Figure S1 **Trypanosoma cruzi infectiveness rates (%) of each species at the tips of the phylogeny and values on the phylogenetic eigenvector 1 (P.E.V. 1) from Agnarsson et al.** (2010). Infectiveness rates were determined as the total positive/total examined*100 in hemoculture or xenodiagnosis tests. Values are scales and normalized around 0 to have a comparable scale.
(PDF)

Table S1 ***Trypanosoma cruzi*** **infection rates in Brazil and species’ diet.**
(PDF)
(PDF)

Table S2 ***Trypanosoma cruzi*** **infectiveness rates (%) of Neotropical wild carnivores from this study and literature records.** Infectiveness rate (INF) for each species based on parasitological tests (hemoculture, xenodiagnosis or fresh blood examination) was calculated by total positive/total examined*100.
(PDF)
