# Supplementary material for: Trypanosoma cruzi Infection in Neotropical Wild Carnivores (Mammalia: Carnivora): At the Top of the T. cruzi Transmission Chain
Source: PLoS One. 2013 Jul 4;8(7):e67463. doi: 10.1371/journal.pone.0067463 (PMC3701642; doi:10.1371/journal.pone.0067463)
Supplement: Figure S1 — Trypanosoma cruzi infectiveness rates (%) of each species at the tips of the phylogeny and values on the phylogenetic eigenvector 1 (P.E.V. 1) from Agnarsson et al. (2010). Infectiveness rates were determined as the total positive/total examined*100 in hemoculture or xenodiagnosis tests. Values are scales and normalized around 0 to have a comparable scale. (PDF) [file pone.0067463.s001.pdf]

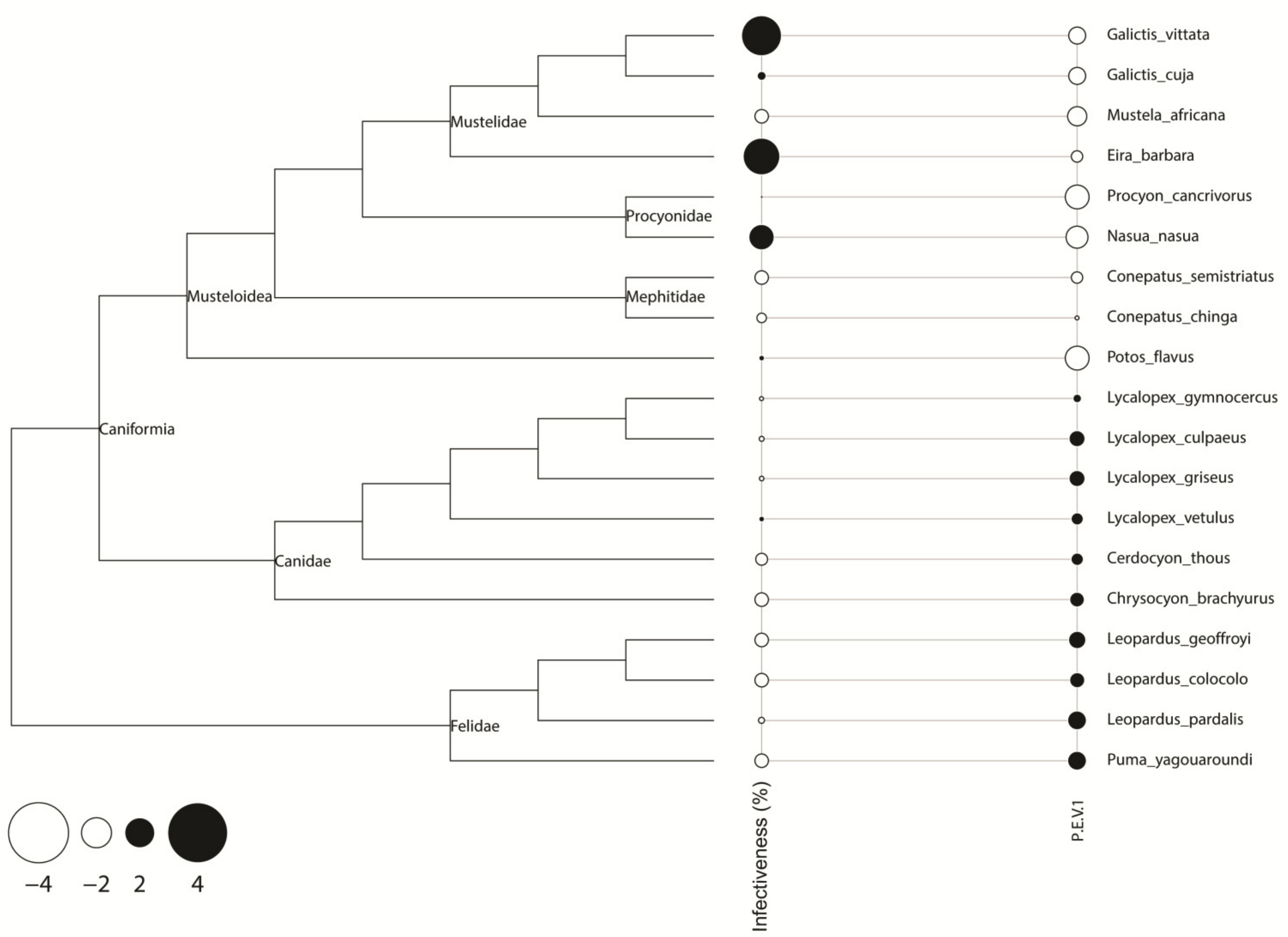

Figure S1. Trypanosoma cruzi infectiveness rates (%) of each species at the tips of the phylogeny and values on the phylogenetic eigenvector 1 (P.E.V. 1) from Agnarsson et al. (2010). Infectiveness rates were determined as the total positive/total examined*100 in hemoculture or xenodiagnosis tests. Values are scales and normalized around 0 to have a comparable scale.

Reference:

Agnarsson I, Kuntner M, May-Collado LJ (2010) Dogs, cats, and kin: a molecular species-level phylogeny of Carnivora. Mol Phylogenet Evol 54: 726-745
