# Supplementary material for: Trypanosoma cruzi Infection in Neotropical Wild Carnivores (Mammalia: Carnivora): At the Top of the T. cruzi Transmission Chain
Source: PLoS One. 2013 Jul 4;8(7):e67463. doi: 10.1371/journal.pone.0067463 (PMC3701642; doi:10.1371/journal.pone.0067463)
Supplement: Table S1 — Trypanosoma cruzi infection rates in Brazil and species’ diet. (PDF) (PDF) [file pone.0067463.s002.pdf]

Supporting Information

Table S1. ***Trypanosoma cruzi*** **infection rates in Brazil and species' diet.**

Species	*T. cruzi* infection P/N (%)[1]	Species diet[2]		Study site	References	
		PM (%)	PI (%)		*T.cruzi* infection	Diet
Cerdocyon thous	9/27 (33)	17.5	25.9	Araguari	this study	Lemos et al 2011
Cerdocyon thous	19/30 (60)	6.4	33.4	Pantanal	this study	Bianchi 2009
Cerdocyon thous	16/42 (38)	6.4	33.4	Pantanal	Herrera et al 2011	Bianchi 2009
Chrysocyon brachyurus	8/39 (21)	24.4	4.3	Canastra	Rocha et al 2013	Queirollo 2007
Chrysocyon brachyurus	11/43 (26)	24.4	4.3	Canastra	this study	Queirollo 2007
Leopardus pardalis	3/10 (30)	46.4	6.7	Pantanal	Herrera et al 2011	Bianchi 2009
Lycalopex vetulus	15/21 (71)	5.5	70.4	Araguari	this study	Lemos et al 2011
Nasua nasua	21/44 (48)	1.5	46.6	Pantanal	this study	Bianchi 2009
Nasua nasua	101/158 (64)	1.5	46.6	Pantanal	Herrera et al. 2008	Bianchi 2009
Nasua nasua	75/140 (54)	1.5	46.6	Pantanal	Alves et al 2011	Bianchi 2009
Procyon cancrivorus	9/12 (75)	3.3	32.2	Pantanal	this study	Gatti 2006

Footnotes:

[1] *T. cruzi* infection based on Indirect Immunofluorescent Antibody Test (IFAT): Positive/Total number of examined (% positive)

[2] Species diet: PM – proportion of mammals; PI – proportion of invertebrates

References - Table S1

Alves FM, Olifiers N, Bianchi RC, Duarte AC, Cotias PM, D'Andrea PS, Gompper ME, Mourao GM, Herrera HM, Jansen AM (2011) Modulating variables of *Trypanosoma cruzi* and *Trypanosoma evansi* transmission in free-ranging coati (*Nasua nasua*) from the Brazilian Pantanal region. Vector Borne Zoonotic Dis 11: 835-841.

Bianchi, R. C. (2009) Ecologia de mesocarnívoros em uma área no Pantanal Central, Mato Grosso do Sul. [Phd thesis]. Corumbá: Federal University of Mato Grosso do Sul. 193 p. Available: http://www.cpap.embrapa.br/teses/online/TSE44.pdf.

Gatti A, Bianchi R, Rosa CRX, Mendes SL (2006) Diet of two sympatric carnivores, *Cerdocyon thous* and *Procyon cancrivorus*, in a restinga area of Espirito Santo State, Brazil. Journal of Tropical Ecology 22: 227–30.

Herrera HM, Rocha FL, Lisboa CV, Rademaker V, Mourao GM, Jansen AM (2011) Food web connections and the transmission cycles of *Trypanosoma cruzi* and *Trypanosoma evansi* (Kinetoplastida, Trypanosomatidae) in the Pantanal Region, Brazil. Trans R Soc Trop Med Hyg 105: 380-387.

Herrera HM, Lisboa CV, Pinho AP, Olifiers N, Bianchi RC, Rocha FL, Mourao GM, Jansen AM (2008) The coati (*Nasua nasua*, Carnivora, Procyonidae) as a reservoir host for the main lineages of *Trypanosoma cruzi* in the Pantanal region, Brazil. Trans R Soc Trop Med Hyg 102: 1133-1139.

Lemos FG, Facure KG, Azevedo FC (2011) A first approach to the comparative ecology of the hoary fox and the crab-eating fox in a fragmented human altered landscape in the Cerrado Biome at Central Brazil. In: Rosalino LM, Gheler-Costa C, editors. Middle-sized carnivores in agricultural landscapes. New York: Nova Sciences Publishers. pp. 143-160.

Queirolo D, Motta-Junior JC (2007) Prey availability and diet of maned wolf in Serra da Canastra National Park, southeastern Brazil. Acta Theriologica 52: 391-402.

Rocha FL, Roque AL, Arrais RC, Santos JP, Lima VD, Xavier SC, Cordeiro-Estrela P, D'Andrea PS, Jansen AM (2013) *Trypanosoma cruzi* TcI and TcII transmission among wild carnivores, small mammals and dogs in a conservation unit and surrounding areas, Brazil. Parasitology 140: 160-170.
