# Supplementary material for: Trypanosoma cruzi Infection in Neotropical Wild Carnivores (Mammalia: Carnivora): At the Top of the T. cruzi Transmission Chain
Source: PLoS One. 2013 Jul 4;8(7):e67463. doi: 10.1371/journal.pone.0067463 (PMC3701642; doi:10.1371/journal.pone.0067463)
Supplement: Table S2 — Trypanosoma cruzi infectiveness rates (%) of Neotropical wild carnivores from this study and literature records. Infectiveness rate (INF) for each species based on parasitological tests (hemoculture, xenodiagnosis or fresh blood examination) was calculated by total positive/total examined*100. (PDF) [file pone.0067463.s003.pdf]

Table S2. *Trypanosoma cruzi* infectiveness rates (%) of Neotropical wild carnivores from this study and literature records. Infectiveness rate (INF) for each species based on parasitological tests (hemoculture, xenodiagnosis or fresh blood examination) was calculated by total positive/total examined*100.

Species[a]	N	POS	NEG	(%)	Site	Country	Reference
**Family CANIDAE**							
Cerdocyon thous	30	0	30	0	Pantanal	Brazil	this study
Cerdocyon thous	3	0	3	0	Canastra	Brazil	Rocha et al. 2013
Cerdocyon thous	42	0	42	0	Pantanal	Brazil	Herrera et al. 2011
Cerdocyon thous	1	0	1	0	Corrientes	Argentina	Bar et al. 1999
Cerdocyon thous	5	0	5	0	Pará	Brazil	Lainson et al. 1979
Cerdocyon thous	5	1	4	20	São Paulo	Brazil	Albuquerque & Barretto 1968
**INF *Cerdocyon thous***	**86**	**1**		**1.2**			
Chrysocyon brachyurus	30	0	30	0	Canastra	Brazil	Rocha et al. 2013
**INF *Chrysocyon brachyurus***	**30**	**0**		**0**			
Lycalopex culpaeus	77	4	77	5	-	Chile	Neghme & Schenone 1962
Lycalopex culpaeus	15	1	14	7	Freirinha	Chile	Whiting 1946
Lycalopex culpaeus	2	1	1	50	Jujuy	Argentina	Mazza 1940
**INF *Lycalopex culpaeus***	**94**	**6**		**6.4**			
Lycalopex griseus	1	0	1	0	Santiago del Estero	Argentina	Wisnivesky-Colli et al 1992
Lycalopex griseus	29	2	27	7	Freirinha	Chile	Whiting 1946
**INF *Lycalopex griseus***	**30**	**2**		**6.7**			
Lycalopex gymnocercus	1	0	1	0	Santiago del Estero	Argentina	Cardinal et al. 2008
Lycalopex gymnocercus	5	0	5	0	Santiago del Estero	Argentina	Ceballos et al. 2006
Lycalopex gymnocercus	3	0	3	0	Corrientes	Argentina	Bar et al. 1999
Lycalopex gymnocercus	1	0	1	0	Santiago del Estero	Argentina	Wisnivesky-Colli et al 1992
Lycalopex gymnocercus	3	0	3	0	Jujuy	Argentina	Schweigmann et al. 1992
Lycalopex gymnocercus	1	1	0	100	Salta	Argentina	Mazza 1940
**INF *Lycalopex gymnocercus***	**14**	**1**		**7.1**			

Species[a]	N	POS	NEG	(%)	Site	Country	Reference
**Family CANIDAE**							
Lycalopex vetulus	6	0	6	0	Canastra	Brazil	Rocha et al. 2013
Lycalopex vetulus	1	1	0	100	Franca	Brazil	Albuquerque & Barretto 1970
**INF *Lycalopex vetulus***	**7**	**1**		**14.3**			
**Family FELIDAE**							
Leopardus colocolo	5	0	5	0	Santiago del Estero	Argentina	Wisnivesky-Colli et al 1992
**INF *Leopardus colocolo***	**5**	**0**		**0**			
Leopardus geoffroyi	2	0	2	0	Chaco Province	Argentina	Alvarado-Otegui et al. 2012
Leopardus geoffroyi	1	0	1	0	Chaco Province	Argentina	Diosque et al. 2004
Leopardus geoffroyi	4	0	4	0	Jujuy	Argentina	Schweigmann et al. 1992
Leopardus geoffroyi	7	0	7	0	Santiago del Estero	Argentina	Wisnivesky-Colli et al 1992
**INF *Leopardus geoffroyi***	**14**	**0**		**0**			
Leopardus pardalis	2	0	2	0	Pantanal	Brazil	this study
Leopardus pardalis	1	1	0	100	Canastra	Brazil	Rocha et al. 2013
Leopardus pardalis	10	0	3	0	Pantanal	Brazil	Herrera et al. 2011
Leopardus pardalis	5	0	5	0	Pará	Brazil	Deane 1961
**INF *Leopardus pardalis***	**18**	**1**		**5.6**			
Puma yagouaroundi	3	0	3	0	Santiago del Estero	Argentina	Wisnivesky-Colli et al 1992
**INF *Puma yagouaroundi***	**3**	**0**		**0**			
**Family MEPHITIDAE**							
Conepatus chinga	15	1	14	7	Santiago del Estero	Argentina	Cardinal et al. 2008
Conepatus chinga	91	1	91	1	Santiago del Estero	Argentina	Ceballos, et al. 2006
Conepatus chinga	1	0	1	0		Paraguay	Yeo et al. 2005
Conepatus chinga	6	0	6	0	Chaco Province	Argentina	Diosque et al. 2004
Conepatus chinga	36	2	34	6	Santiago del Estero	Argentina	Wisnivesky-Colli et al 1992
Conepatus chinga	49	2	47	4	Santiago del Estero	Argentina	Pietrokovsky et al. 1991
Conepatus chinga	3	0	3	0	Til-til	Chile	Whiting 1946
**INF *Conepatus chinga***	**201**	**6**		**3.0**			

Species[a]	N	POS	NEG	(%)	Site	Country	Reference
**Family MEPHITIDAE**							
Conepatus semistriatus	2	0	2	0	Canastra	Brazil	Rocha et al. 2013
**INF *Conepatus semistriatus***	**2**	**0**		**0**			
**Family MUSTELIDAE**							
Eira barbara	5	1	4	20	São Paulo	Brazil	Barretto & Ribeiro 1972
Eira barbara	2	1	1	50	Mato Grosso	Brazil	Deane 1964
Eira barbara	4	2	2	50	Pará	Brazil	Deane 1961
Eira barbara	4	1	4	25	Pará	Brazil	Rodrigues & Melo 1942
Eira barbara	1	1	0	100	Jujuy	Argentina	Mazza 1940
**INF *Eira barbara***	**16**	**6**		**37.5**			
Galictis cuja	1	0	1	0	Corrientes	Argentina	Bar et al. 1999
Galictis cuja	2	0	2	0	Jujuy	Argentina	Schweigmann et al. 1992
Galictis cuja	1	1	0	100	Santiago del Estero	Argentina	Wisnivesky-Colli et al 1992
Galictis cuja	14	2	12	14	São Paulo	Brazil	Ferriolli & Barretto 1969
**INF *Galictis cuja***	**18**	**3**		**16.7**			
Galictis vittata	1	1	0	100	Sumidouro/RJ	Brazil	Lisboa et al. 2009
Galictis vittata	1	1	0	100	São Paulo	Brazil	Barretto & Albuquerque 1971
Galictis vittata	3	0	3	0	Pará	Brazil	Deane 1961
**INF *Galictis vittata***	**5**	**2**		**40**			
Mustela Africana	4	0	4	0	Pará	Brazil	Deane 1961
**INF *Mustela Africana***	**4**	**0**		**0**			
**Family PROCYONIDAE**							
Nasua nasua	66	19	49	29	Pantanal	Brazil	this study
Nasua nasua	140	53	87	38	Pantanal	Brazil	Alves et al. 2011
Nasua nasua	158	33	125	21	Pantanal	Brazil	Herrera et al. 2008
Nasua nasua	18	7	11	39	Pará	Brazil	Lainson et al. 1979
Nasua nasua	5	1	0	20	São Paulo	Brazil	Ferriolli & Barretto 1968
Nasua nasua	2	0	2	0	Pará	Brazil	Deane 1961

Species[a]	N	POS	NEG	(%)	Site	Country	Reference
**Family PROCYONIDAE**							
Nasua nasua	2	0	2	0	Pará	Brazil	Rodrigues & Melo 1942
**INF *Nasua nasua***	**391**	**113**		**28.9**			
Potos flavus	2	1	0	50	Bajo Colima	Colombia	Travi et al. 1994
Potos flavus	2	0	2	0		Guiana Francesa	Deurere et al. 2001
Potos flavus	1	0	1	0	Pará	Brazil	Lainson et al. 1979
Potos flavus	2	0	2	0	Pará	Brazil	Rodrigues & Melo 1942
**INF *Potos flavus***	**7**	**1**		**14.3**			
Procyon cancrivorus	13	2	11	15	Pantanal	Brazil	this study
Procyon cancrivorus	2	0	2	0	Chaco Province	Argentina	Alvarado-Otegui et al. 2012
Procyon cancrivorus	4	1	3	25	São Paulo	Brazil	Barretto & Ferriolli 1970
Procyon cancrivorus	12	0	12	0	Pará	Brazil	Deane 1961
**INF *Procyon cancrivorus***	**31**	**3**		**9.7**			

Footnote:
[a] We adopted Wilson & Reeder (2005) for taxonomic reference; thus, host species names reported in this table not always correspond to the original paper.

References Table S2

Albuquerque RD, Barretto MP (1968) Studies on wild reservoirs and vectors of "*Trypanosoma cruzi*." XXX: natural infection of the bush dog, "Cerdocyon thous azarae" (Wied, 1824) by "*T. cruzi*". Rev Bras Biol 28: 457-468.

Albuquerque RD, Barretto MP (1970) Studies on wild reservoirs and vectors of *Trypanosoma cruzi*. XLIV. Natural infection of the field fox, *Dusicyon (Lycalopex) vetulus* (Lung, 1842) by *T. cruzi*. Rev Inst Med Trop Sao Paulo 12: 375-382.

Alvarado-Otegui JA, Ceballos LA, Orozco MM, Enriquez GF, Cardinal MV, Cura C, Schijman AG, Kitron U, Gurtler RE (2012) The sylvatic transmission cycle of Trypanosoma cruzi in a rural area in the humid Chaco of Argentina. Acta Trop 124: 79-86.

Alves FM, Olifiers N, Bianchi RC, Duarte AC, Cotias PM, D'Andrea PS, Gompper ME, Mourao GM, Herrera HM, Jansen AM (2011) Modulating variables of *Trypanosoma cruzi* and Trypanosoma evansi transmission in free-ranging Coati (*Nasua nasua*) from the Brazilian Pantanal region. Vector Borne Zoonotic Dis 11: 835-841.

Bar ME, Alvarez BM, Oscherov EB, Damborsky MP, Jorg ME (1999) Contribución al conocimiento de los reservorios del *Trypanosoma cruzi* (Chagas, 1909) en la Provincia de Corrientes, Argentina. Rev. Soc. Bras. Med. Trop. 32(3): 271–276.

Barretto MP, Albuquerque RD (1971) Studies on reservoirs and wild vectors of *Trypanosoma cruzi*. XLVII. Natural infection of the mustelid, *Galictis vittata braziliensis* (Thunberg, 1820) by *T. cruzi*. Rev Inst Med Trop Sao Paulo 13: 346-351.

Barretto MP, Ferriolli FF (1970) Studies on wild reservoirs and vectors of *Trypanosoma cruzi*. XXXIX: Natural infection of *Procyon cancrivorus nigripes* Mivart, 1885, by *T. cruzi*. Rev Bras Biol 30: 431-438.

Barretto MP, Ribeiro RD (1972) Studies on wild reservoirs and vectors of *Trypanosoma cruzi*. LI. Natural infection of the mustelid, *Eira barbara barbara* (Lin., 1758) by *T. cruzi*. Rev Bras Biol 32: 413-418.

Cardinal MV, Lauricella MA, Ceballos LA, Lanati L, Marcet PL, Levin MJ, Kitron U, Gurtler RE, Schijman AG (2008) Molecular epidemiology of domestic and sylvatic *Trypanosoma cruzi* infection in rural northwestern Argentina. Int J Parasitol 38: 1533-1543.

Ceballos LA, Cardinal MV, Vazquez-Prokopec GM, Lauricella MA, Orozco MM, Cortinas R, Schijman AG, Levin MJ, Kitron U, Gurtler RE (2006) Long-term reduction of *Trypanosoma cruzi* infection in sylvatic mammals following deforestation and sustained vector surveillance in northwestern Argentina. Acta Trop 98: 286-296.

Deane LM (1961) Tripanosomídeos de mamíferos da região amazônica. I – Alguns flagelados encontrados no sangue de mamíferos silvestres do Estado do Pará. Rev Inst Med Trop Sao Paulo 3: 15-28.

Deane LM (1964) Animal reservoirs of *Trypanosoma cruzi* in Brazil. Rev Bras Malariol Doencas Trop 16: 27-48.

Dereure J, Barnabé C, Vié JC, Madélenat F, Raccurt C. (2001) Trypanosomatidae from wild mammals in the neotropical rainforest of French Guiana. Annals of tropical medicine and parasitology 95(2):157–66.

Diosque P, Padilla AM, Cimino RO, Cardozo RM, Negrette OS, Marco JD, et al. (2004) Chagas disease in rural areas of Chaco Province, Argentina: epidemiologic survey in humans, reservoirs, and vectors. Amer J Trop Med Hyg 71(5): 590-593.

Ferriolli FF, Barretto MP (1968) Studies on wild reservoirs and vectors of *Trypanosoma cruzi*. XXIX. Natural infection of *Nasua nasua* solitaria Schinz 1821 by *T. cruzi*. Rev Inst Med Trop Sao Paulo 10: 354-363.

Ferriolli FF, Barretto MP (1969) Studies on wild reservoirs and vectors of *Trypanosoma cruzi*. XXXV. Natural infection of the ferret *Galictis cuja furax* (Thomas, 1907) by *T. cruzi*. Rev Inst Med Trop Sao Paulo 11: 264-273.

Herrera HM, Lisboa CV, Pinho AP, Olifiers N, Bianchi RC, Rocha FL, Mourao GM, Jansen AM (2008) The coati (*Nasua nasua*, Carnivora, Procyonidae) as a reservoir host for the main lineages of *Trypanosoma cruzi* in the Pantanal region, Brazil. Trans R Soc Trop Med Hyg 102: 1133-1139.

Herrera HM, Rocha FL, Lisboa CV, Rademaker V, Mourao GM, Jansen AM (2011) Food web connections and the transmission cycles of *Trypanosoma cruzi* and *Trypanosoma evansi* (Kinetoplastida, Trypanosomatidae) in the Pantanal Region, Brazil. Trans R Soc Trop Med Hyg 105: 380-387.

Lainson R, Shaw JJ, Fraiha H, Miles MA, Draper CC (1979) Chagas's Disease in the Amazon Basin: 1. *Trypanosoma cruzi* infections in silvatic mammals, triatomine bugs and man in the State of Para, north Brazil. Trans R Soc Trop Med Hyg 73: 193-204.

Lisboa CV, Xavier SC, Herrera HM, Jansen AM (2009) The ecology of the *Trypanosoma cruzi* transmission cycle: Dispersion of zymodeme 3 (Z3) in wild hosts from Brazilian biomes. Vet Parasitol 165: 19-24.

Mazza S (1940) Otros mamíferos infectados naturalmente por Schizotrypanum cruzi, o cruzi similes en provincias de Jujuy y Salta. Misión de estudios de Patología Regional Argentina - M.E.P.R.A. 119–34.

Neghme A, Schenone H (1962) Enfermedad de Chagas en Chile: viente años de investigación. Ann Congr Int Doença de Chagas 3: 1069-1105.

Pietrokovsky SM, Schweigmann NJ, Riarte A, Alberti A, Conti O, Montoya S, Wisnivesky-Colli C (1991) The skunk *Conepatus chinga* as new host of *Trypanosoma cruzi* in Argentina. J Parasitol 77: 643-645.

Rocha FL, Roque AL, Arrais RC, Santos JP, Lima VD, Xavier SC, Cordeiro-Estrela P, D'Andrea PS, Jansen AM (2013) *Trypanosoma cruzi* TcI and TcII transmission among wild carnivores, small mammals and dogs in a conservation unit and surrounding areas, Brazil. Parasitology 140: 160-170.

Rodrigues BA, Melo GB. (1942) Contribuição ao estudo da Tripanosomiase americana. Mem Inst Oswaldo Cruz. 37(1):77–93.

Schweigmann NJ, Alberti A, Pietrokovsky SM, Conti O, Montoya S, Wisnivvesky-Colli C (1992) A new host of *Trypanosoma cruzi* from Jujuy, Argentina: *Octodontomys gliroides* (Gervais & D'orbigny, 1844) (Rodentia, Octodontidae). Mem Inst Oswaldo Cruz. 87(2): 217–20.

Travi BL, Jaramillo C, Montoya J, Segura I, Zea A, Goncalves A, Velez ID (1994) *Didelphis Marsupialis*, an important reservoir of *Trypanosoma (Schizotrypanum) cruzi* and *Leishmania (Leishmania) chagasi* in Colombia. Amer J Trop Med Hyg 50: 557-565.

Whiting C (1946) Contribuición al estudio de las reservas de parasitos de la enfermedad de Chagas en Chile. Primeiros hallazgos en Chile de mamiferos silvestres infestados por *Trypanosoma cruzi*. Rev Chil Hig Med Prev 8: 69-100.

Wilson DE, Reeder DM(editors) (2005) Mammal Species of the World. A Taxonomic and Geographic Reference. Johns Hopkins University Press. 2142 p.

Wisnivesky-Colli C, Schweigmann NJ, Alberti A, Pietrokovsky SM, Conti O, Montoya S, Riarte A, Rivas C (1992) Sylvatic American trypanosomiasis in Argentina. *Trypanosoma cruzi* infection in mammals from the Chaco forest in Santiago del Estero. Trans R Soc Trop Med Hyg 86: 38-41.

Yeo M, Acosta N, Llewellyn M, Sanchez H, Adamson S, Miles GA, Lopez E, Gonzalez N, Patterson JS, Gaunt MW, de Arias AR, Miles MA (2005) Origins of Chagas disease: *Didelphis* species are natural hosts of *Trypanosoma cruzi* I and armadillos hosts of *Trypanosoma cruzi* II, including hybrids. Int J Parasitol 35: 225-233.
